# Supplementary material for: Arsenic Oxidation and Removal from Water via Core–Shell MnO2@La(OH)3 Nanocomposite Adsorption
Source: Int J Environ Res Public Health. 2022 Aug 26;19(17):10649. doi: 10.3390/ijerph191710649 (PMC9518204; doi:10.3390/ijerph191710649)
Supplement: Supplementary file 1 [file ijerph-19-10649-s001.zip › ijerph-1821963-supplementary.pdf]

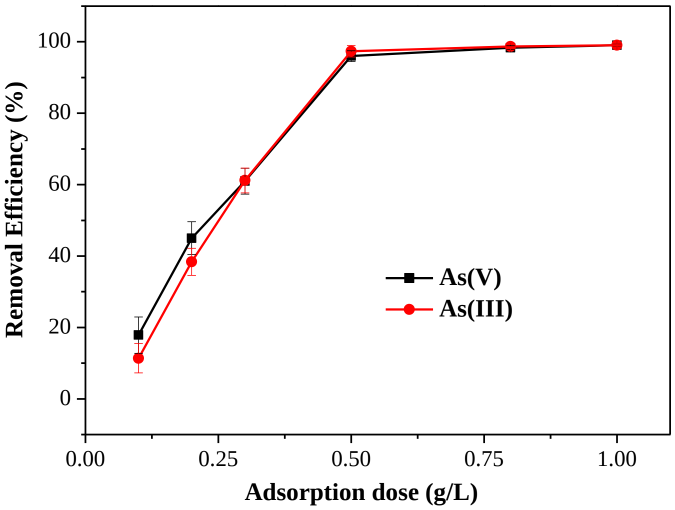

Fig. S1. Effect of MnO_2@$La(OH)_3$ dosage on arsenic removal at pH 4.0. Experimental conditions: adsorbent dosage, 0.5 g/L; initial As concentration, 65 mg/L.

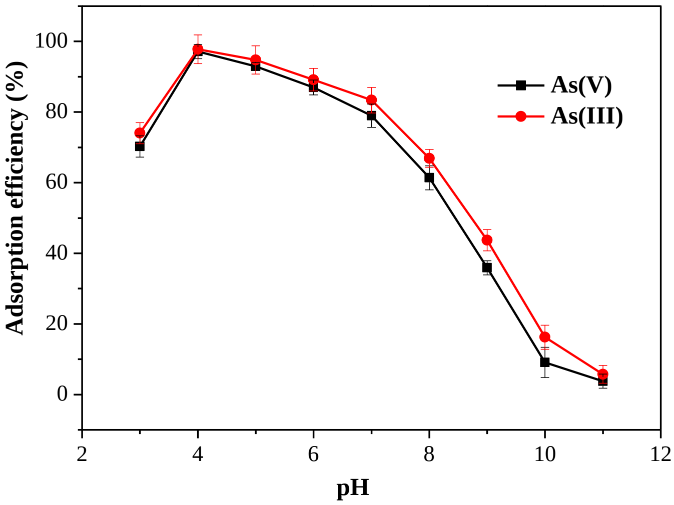

Fig. S2. Comparison of the effect of solution pH on As(V) and As(III) removal. Experimental conditions: adsorbent dosage, 0.5 g/L; initial As concentration, 65 mg/L.

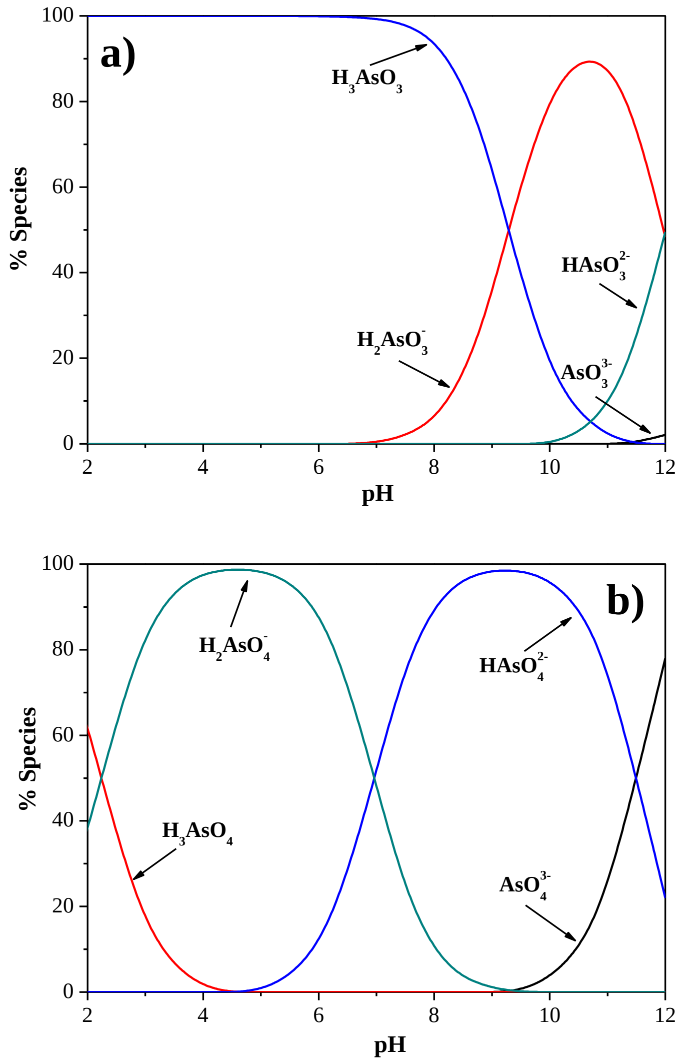

Fig. S3. The distribution of arsenic species under different pH: a) As(III), and b) As(V).
